# DNA methylation profiling identifies the HOXA11 gene as an early diagnostic and prognostic molecular marker in human lung adenocarcinoma

**DOI:** 10.18632/oncotarget.16528

**Published:** 2017-03-23

**Authors:** Qun Li, Chang Chen, Xiaohui Ren, Weihong Sun

**Affiliations:** ^1^ Key Laboratory of Stem Cell Biology, Institute of Health Sciences, Shanghai Institutes for Biological Sciences, Chinese Academy of Sciences, Shanghai Jiao Tong University School of Medicine, Shanghai, 200031, China; ^2^ The State Key Laboratory of Medical Genomics, Shanghai Key Laboratory of Hypertension, Ruijin Hospital, Shanghai Institute of Hypertension, Shanghai Jiao Tong University School of Medicine, Shanghai, 200025, China; ^3^ Department of Orthodontics, The First Affiliated Hospital of Zhengzhou University, Stomatological College Zhengzhou University, Zhengzhou, 450052, China

**Keywords:** HOXA11, hypermethylation, lung adenocarcinoma, adenocarcinoma in situ, prognosis

## Abstract

DNA hypermethylation plays important roles in carcinogenesis by silencing key genes. The goal of our study was to identify pivotal genes using MethyLight and assessed their diagnostic and prognostic values in lung adenocarcinoma (AD). In the present study, we detected DNA methylation at sixteen loci promoter regions in twenty one pairs of primary human lung AD tissues and adjacent non-tumor lung (AdjNL) tissues using the real-time PCR (RT-PCR)-based method MethyLight. By comparing the sixteen analyzed loci in lung AD tissues and AdjNL and non-tumor (NL) tissues, we found that, among the six genes identified with hypermethylation, the HOXA11, CDKN2A-EX2 and EYA4 genes showed highly promising DNA hypermethylation diagnostic markers in the lung AD tissues. Moreover, comparing lung AD tissues (> 2 cm in diameter) to the AdjNL or AD *in situ* (AIS) tissues by RT-qPCR and immunohistochemistry revealed that HOXA11 expression was significantly increased. A further study showed that HOXA11 expression was controlled by methylation in the promoter region in human lung tumor cell lines. Aberrant hypermethylation and the methylation-induced down-regulation of HOXA11 may promote tumor progression. Our results suggested that HOXA11 might be a diagnostic and prognostic marker in patients with lung AD.

## INTRODUCTION

The morbidity and mortality of lung cancer is increasing most rapidly these years in China. In histology, lung cancers are classified into two main types, including non-small cell lung carcinoma (NSCLC) and small cell lung carcinoma (SCLC). The three major subtypes of NSCLC are AD, squamous-cell carcinoma and large-cell carcinoma. Among them, AD is the remarkable histological subtype and its incidence is raising [1]. The 5-year survival rate of AD is about 15% [2], normally, its mortality rate is high, because the early diagnosis is difficult and its progress is very quickly. Although some clinicopathological features, including tumor size, vascular invasion and histopathological grade, have been shown to be prognostic factors for lung cancer after surgical resection, its prognostic molecular markers have not been well documented. Therefore, the identification of diagnostic and prognostic molecular markers is scientifically and clinically significant. These markers may help reveal the mechanisms of lung AD, serve as potential therapeutic targets and predict patient prognosis.

On the basis of its clinicopathological features, AD has been considered to develop into invasive carcinoma through atypical adenomatous hyperplasia (AAH), AIS, and minimally invasive AD [3]. In 1999, the histological classification of lung cancer was renewed by the World Health Organization, both bronchioloalveolar carcinoma (BAC) and AAH were added as a very early stage of AD (*AIS*) [4]. Therefore, they were considered as early manifestation of AD. Furthermore, Small AD (two centimeters or less in diameter) can be divided into two groups, including replacement type AD and Non-replacement type AD. Each group can be further subdivided into three types, respectively. The subtypes of replacement type AD contain Localized bronchioloalveolar carcinoma (LBAC, type A), LBAC with foci of collapse of alveolar structure (type B) and LBAC with foci of active fibroblastic proliferation (type C), the subtypes of Non-replacement type AD incorporate poorly differentiated AD (type D), tubular AD (type E) and papillary AD with compressive and destructive growth (type F) [5]. The above type A, B and C are considered to be early stage of AD. Patients with type A and B have a very good prognosis, and the 5-year survival rate is 100% [6]. The change of DNA methylation pattern is the best features and the easiest quantifiable epigenetic alteration in lung AD [7]. It is possible that DNA methylation detection is used for cancer screening. In most of the previous studies, a candidate gene approach was used for DNA methylation testing in lung cancer. DNA hypermethylation at promoter region indicates much probably as a cancer-specific marker, it can complement visual tools for lung cancer screening, such as chest radiography and spiral CT, thereby improving early diagnosis. DNA methylation plays an important role in carcinogenesis because it leads to the silencing of many pivotal genes, including tumor suppressor genes [8]. DNA hypermethylation in promoter region occures in all cancers, which can induce the downstream gene silence through the methylation of CpG islands close to the transcriptional initiation sites of genes. Among many DNA molecular markers, DNA hypermethylation is a great promise epigenetic alteration marker.

In this study, we focused on lung AD and identified pivotal hypermethylated genes by using the MethyLight method [9]. We chose 16 loci that showed hypermethylation in promoter region of lung and other kinds of cancers reported by other researchers, and these genes played important roles in transcription, tumor suppressor, apoptosis and cell cycle modulation (Table 1). We found that 6 genes showed hypermethylation in human lung AD tissues. Then, among the 6 hypermethylated genes, we screened for potential prognosis-related genes using AIS tissues and AD tissues. We discovered that HOXA11 was hypermethylated at the early stage of human lung AD and further investigated its diagnostic and prognostic value.

**Table 1:** Gene name and function of the 16 loci studied

Official symbol	Official Full Name^a^	Function^a^
CDH13	cadherin 13	Cell-cell adhesions.
CDKN2A	cyclin-dependent kinase inhibitor 2A	Tumor suppressor. Cell cycle control. involved in proliferation and apoptosis
CDX2	caudal type homeobox 2	Transcriptional regulation. Involved in cell growth and differentiation.
CYP2C35	cytochrome P450 2C35	Unknown.
EYA4	EYA transcriptional coactivator and phosphatase 4	Transcription regulation. Involved in development
HOXA1	homeobox A1	Transcription factor. Involved in development
HOXA11	homeobox A11	Transcription factor.
NEUROD1	neurogenic differentiation 1	Transcriptional activator. Involved in differentiation.
NEUROD2	neurogenic differentiation 2	Transcriptional activity. Involved in development
ONECUT2	one cut homeobox 2	Transcriptional factor.
OPCML	opioid binding protein/cell adhesion molecule-like	Involve in cell contact
PTPRN2	protein tyrosine phosphatase, receptor type, N polypeptide 2	Regulate a variety of cellular processes including cell growth, differentiation, mitotic cycle, and oncogenic transformation
RASSF1	Ras association (RalGDS/AF-6) domain family member 1	Potential tumor suppressor. Involved in apoptosis, proliferation, cell cycle progression.
SFRP1	Secreted frizzled-related protein 1	Epigenetic silencing of SFRP genes leads to deregulated activation of the Wnt-pathway
TMEFF2	transmembrane protein with EGF-like and two follistatin-like domains 2	Down-regulated in tumor cell lines.
TWIST1	Twist family bHLH transcription factor 1	Transcription factor. Involved in differentiation.

^a^Approved gene name and function from National Center for Biotechnology Information(NCBI).

website http://www.ncbi.nlm.nih.gov/

## RESULTS

### DNA methylation profile in lung AD tissues by MethyLight analysis

To identify aberrantly methylated genes in lung AD, we selected the MethyLight method to check the methylation status of promoter regions in lung AD tissues compared to that in AdjNL and control NL tissues. Sixteen loci (Table 1) were chosen in this study. These loci are in the promoter region and have rich CpG islands, which are the part of key factors in tumor suppressor, cell cycle regulation, apoptosis and transcription (Table 1).

**Figure 1:**
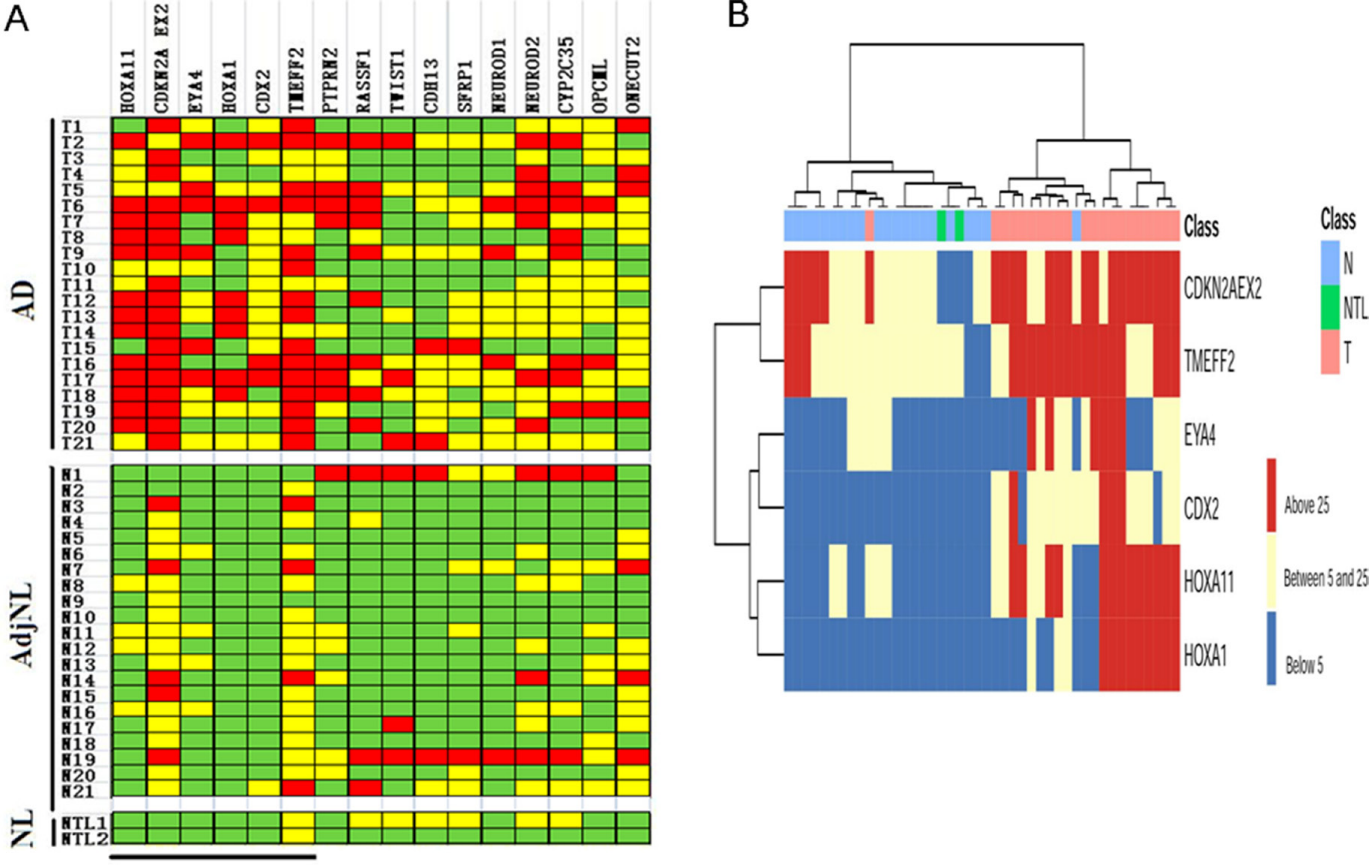
DNA methylation profile in lung AD tissues by MethyLight analysis (**A**) Graphic representation of PMR values obtained from 16 loci in AD, AdjNL and NL samples. Samples are indicated on the left (the same number indicates that the sample is from the same patient) and loci are indicated on the top. PMR values have been categorized as colored boxes denoting DNA methylation above the median (PMR > 25, red), below the median (5 < PMR < 25, yellow) and no detectable DNA methylation (PMR < 5, green). The black bar at the bottom indicates loci showing statistically significant differences in DNA methylation levels between AD and AdjNL. (**B**) Two-dimensional hierarchical clustering of samples and the top 6 loci based on DNA methylation data. DNA methylation levels are indicated by the color shown on the right side. The ward hierarchical clustering method was used to categorize the AD, AdjNL and NL samples. Samples are indicated on the top line, with AD in pink, AdjNL samples in green, and NL samples in blue.

We analyzed the DNA methylation from 16 loci in 21 paired human lung AD and AdjNL tissues and 2 NL tissues, the results were illustrated in Figure 1A. DNA methylation was presented by using PMR and then visualized by using color coding as follows: DNA methylation level above the median (PMR > 25, red), below the median (5 < PMR < 25, yellow) and no detectable DNA methylation (PMR < 5, green). A comparison of the AD and AdjNL tissues was shown in Figure 1A, demonstrating that the six loci (HOXA11, CDKN2A EX2, EYA4, HOXA1, CDX2 and TWEFF2) showed highly significant hypermethylation in lung AD (*p* < 0.01), particularly in the following loci: HOXA11 (*p* = 6.86E-05), CDKN2A EX2 (*p* = 1.85E-04), and EYA4 (*p* = 8.41E-04). We also analyzed the top three markers in all samples and samples based on gender, stage and Noguchi classification (Table 2). The results showed that HOXA11, CDKN2A EX2 and EYA4 were hypermethylated even in AIS samples.

**Table 2:** Performance of top three markers in total samples and samples based on gender, stage and noguchi classification

	Median PMR,Tumor	Median PMR.AdjNL	*p*-value^a^
**Total samples**	*n* = 21	*n* = 21	
HOXA11	48.80	3.03	**6.9E-05**
CDKN2A	48.90	18.10	**1.8E-04**
EYA4	18.98	2.52	**8.4E-04**
**GENDER**			
**Femal**	*n* = 12	*n* = 12	
HOXA11	38.13	1.36	**3.5E-03**
CDKN2A	42.97	20.32	**5.9E-03**
EYA4	13.41	1.85	**1.4E-02**
**Meal**	*n* = 9	*n* = 9	
HOXA11	63.02	5.09	**2.3E-03**
CDKN2A	56.81	11.78	**2.4E-03**
EYA4	26.40	3.01	**1.2E-02**
**STAGE^b^**			
**StageIA**	*n* = 15	*n* = 15	
HOXA11	44.59	2.69	**2.2E-03**
CDKN2A EX2	52.19	21.45	**4.7E-03**
EYA4	15.73	2.59	**9.6E-03**
**StageIB**	n = 2	*n* = 2	
HOXA11	76.67	2.93	4.2E-01
CDKN2A EX2	33.21	7.07	2.8E-01
EYA4	42.55	0.99	9.8E-02
**StageIIA/IIB/IIIA**	*n* = 4	*n* = 4	
HOXA11	50.66	4.34	**6.8E-03**
CDKN2A EX2	44.41	11.04	**3.7E-02**
EYA4	19.38	3.00	2.8E-01
**Noguchi classification**			
**TypeA,B**	*n* = 15	*n* = 15	
HOXA11	48.43	2.95	**8.8E-04**
CDKN2A EX2	51.65	20.38	**4.5E-03**
EYA4	13.96	2.64	**2.1E-02**
**> 2cm**	*n* = 6	*n* = 6	
HOXA11	49.73	3.22	5.7E-02
CDKN2A EX2	42.02	12.40	**3.6E-03**
EYA4	31.54	2.23	**1.6E-02**

We checked the relevance between the top six hypermethylated genes and the three different tissues (lung AD, AdjNL and NL tissues) by using two-dimensional hierarchical clustering analysis (Figure 1B). Six loci, HOXA1, HOXA11, CDX2, EYA4, TWEFF2 and CDKN2A EX2, clustered together (left), showing significant methylation in the lung AD (top left) samples.

**Figure 2:**
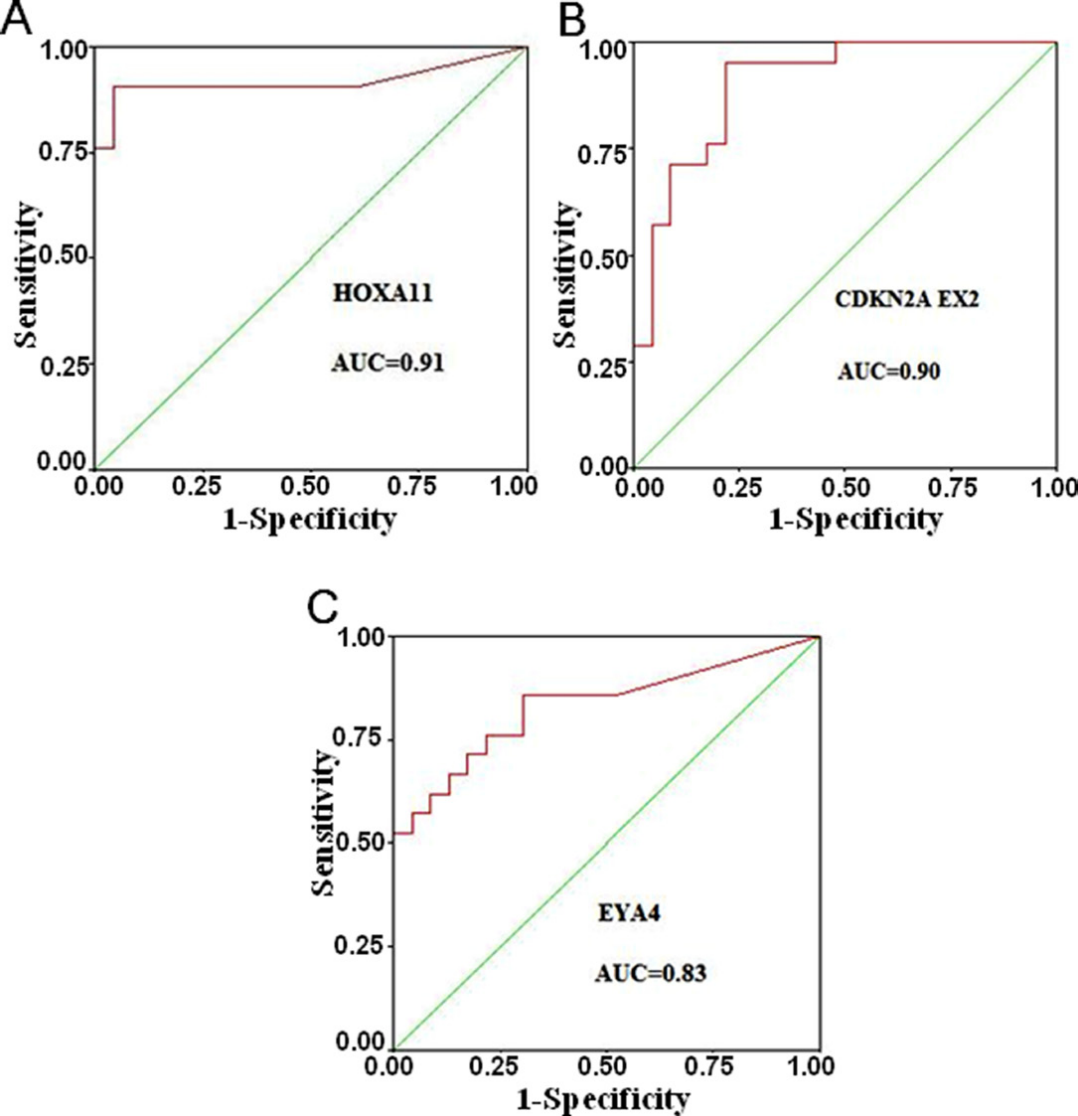
Receiver operating characteristic (ROC) curves for the three top markers The AUC (This indicates of marker performance that would be 1 for a marker showing 100% specificity and sensitivity) is 0.83–0.91 for the three top loci (**A**) HOXA11, (**B**) CDKN2A EX2 and (**C**) EYA4.

We further analyzed the receiver operating characteristic (ROC) curve using the SPSS statistics software. As shown in Figure 2, the area under the curve (AUC, which indicates a marker performance), that is equal to 1 for a marker meaning 100% specificity and sensitivity, AUC is 0.83–0.91 for the three top loci as follows: HOXA11 (AUC = 0.91), CDKN2A EX2 (AUC = 0.90), and EYA4 (AUC = 0.83). These three genes, particularly the HOXA11 and CDKN2A EX2 genes, can much probably be taken as new DNA hypermethylation markers for early diagnosis in lung AD. Thus, these data suggested that HOXA11 and CDKN2A EX2 might be DNA hypermethylation markers for lung AD.

### Expression of HOXA11, EYA4 and CDKN2A in human AD and AdjNL tissues

**Figure 3:**
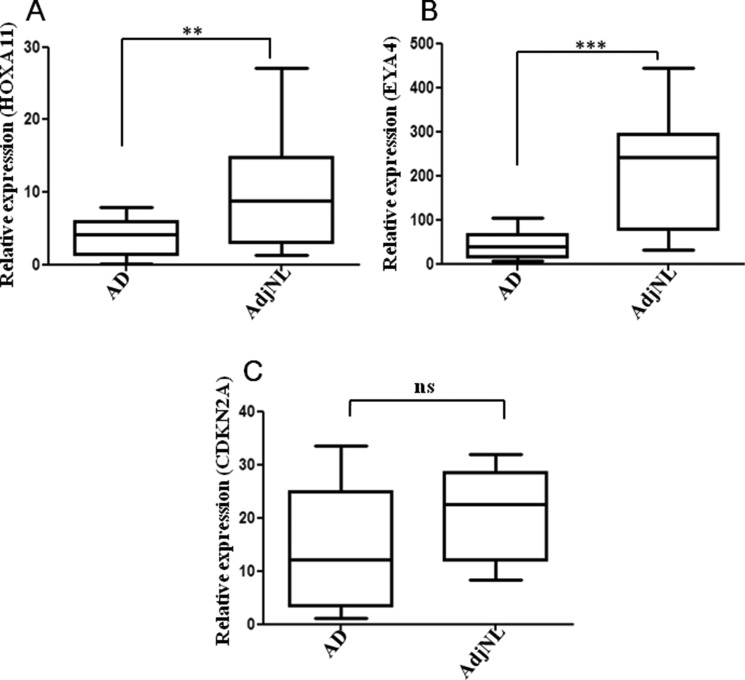
Real-time qPCR analysis of the (**A**) HOXA11, (**B**) EYA4 and (**C**) ENKN2A expression in AD and AdjNL tissues. All measurements are shown relative to the expression level of the GAPDH gene. Bars show the means ± SD. Difference was statistically significant (*p < 0.01. **p < 0.001. ***p < 0.0001). (AD, AdjNL, 30 pairs tissues).

We investigated the top three hypermethylation genes (HOXA11, EYA4 and CDKN2A) mRNA expression using RT-qPCR in lung AD tissues and AdjNL tissues. The relative mRNA expression of HOXA11 and EYA4 was significantly higher in AdjNL than that in lung AD tissues (Figure 3A and 3B), whereas CDKN2A expression was not significantly different (Figure 3C). Our results revealed that the promoter hypermethylation of HOXA11 and EYA4 could down-regulate their mRNA expression.

### Effect of HOXA11 expression on 5-AZA-dC induced-lung AD cell lines

**Figure 4:**
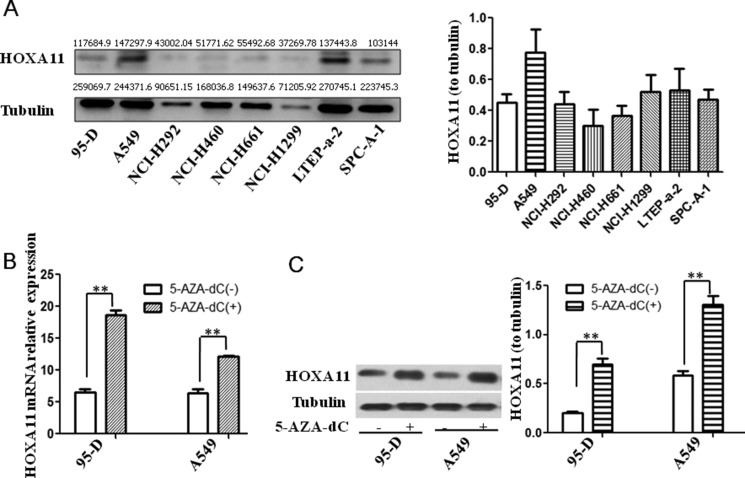
HOXA11 protein expression in lung cancer cell lines and effects of 5-Aza-dC on demethylation and re-expression of silenced HOXA11 (**A**) HOXA11 protein expression in lung cancer cell lines. We marked the gray value on each WB band using ImageJ analyzing. (**B**) Re-expression of silenced HOXA11 mRNA was examined by qPCR in lung AD cell lines (95D and A549) after the treatment of the cells with 5-AZA-dC for 48 hrs. (**C**) Re-expression of silenced HOXA11 protein was detected by Western blot analysis in lung AD cell lines (95D and A549) after the treatment of the cells with 5-AZA-dC for 48 hrs. (**p < 0.001).

We chose eight human lung cancer cell lines from our cell bank to detect HOXA11 protein expression by a western blot estimation of the HOXA11 protein band intensity normalized to Tubulin (Figure 4A). Then, we selected the two lung AD cell lines 95D and A549 and stimulated these cell lines with or without 5-AZA-dC; we found that the HOXA11 mRNA expression and protein release were significantly restored in both cell lines after the 5-AZA-dC stimulation (Figure 4B, 4C). Our results revealed that 5-AZA-DC could restore HOXA11 protein expression.

### Comparison of HOXA11 gene expression in lung AD, AIS and AdjNL tissues by immunohistochemical staining

**Figure 5:**
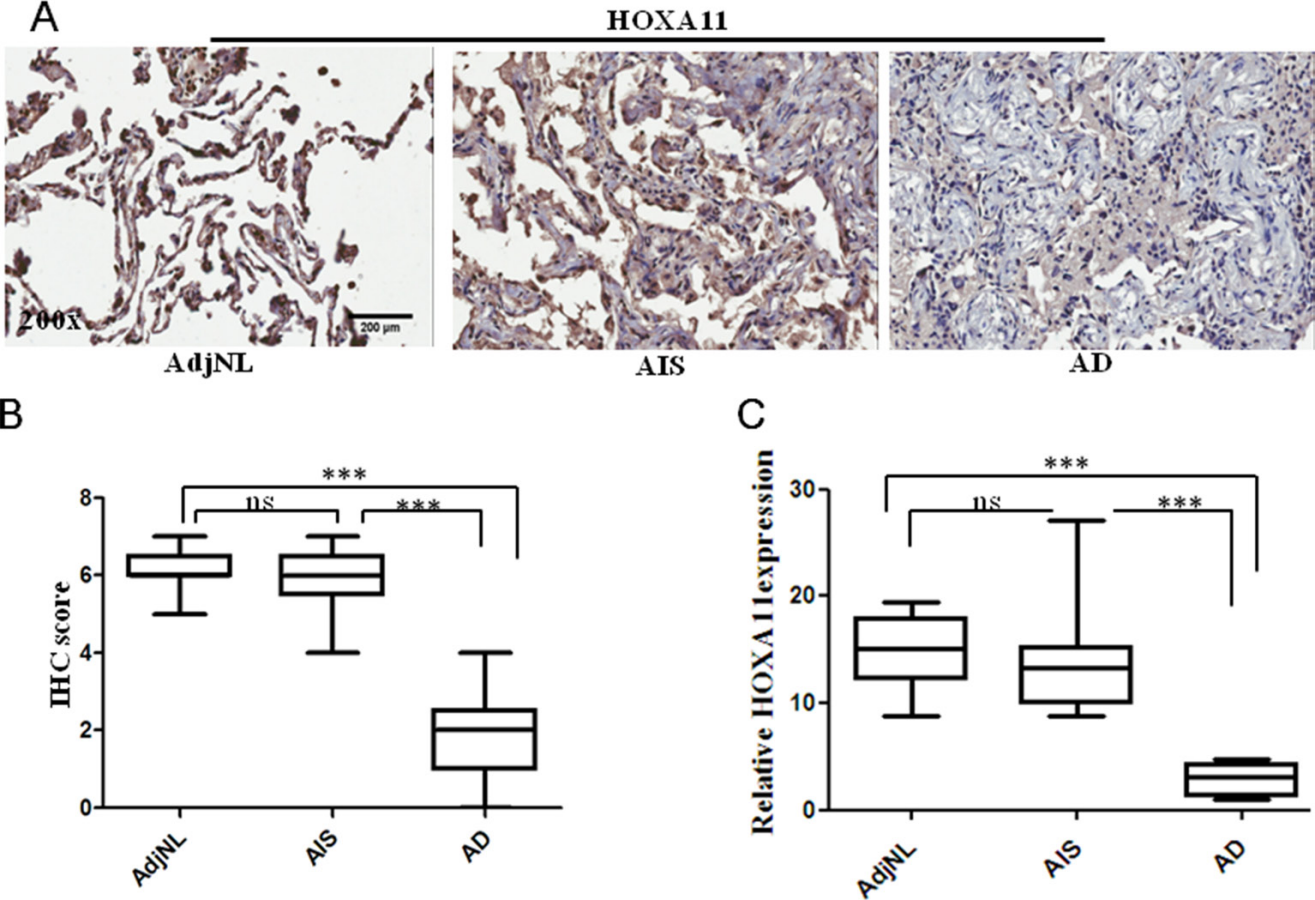
Immunohistochemical (IHC) staining of HOXA11 protein in lung tissues (**A**) A strong staining in AdjNL. A strong staining in AIS. A weak staining in AD. (**B**) Boxplot of the HOXA11 IHC scores illustrated that AD had a lower HOXA11 protein level than AIS and AdjNL (***p < 0.0001). (**C**) Relative expression of HOXA11 in human lung AdjNL, AIS and AD tissues. Bars show the means ± SD. Difference was statistically significant (*p < 0.01. **p < 0.001. ***p < 0.0001). Original magnification, 200x. Scale bar, 200 μm.

To estimate the clinical significance of HOXA11, we investigated the HOXA11 protein expression in TMA from 160 lung cancer patients by using immunohistochemical analysis. For the histological analysis, sections taken from human lung AD (> 2 cm), AIS and AdjNL tissues were stained for the HOXA11 protein. We found that HOXA11 protein expression was lower in lung AD tissues than in the AIS and AdjNL tissues. However, there were no significant differences between the AIS and AdjNL tissues (Figure 5A, 5C). HOXA11 protein expression was mainly observed in the nucleus (Figure 5A). In addition, HOXA11 protein expression had an inverse relationship with the tumor clinicopathological type, with lower HOXA11 protein levels in AD tissues compared to those in AIS tissues. The HOXA11 IHC scores illustrated that the AD tissues had a lower HOXA11 protein level than the AIS and AdjNL tissues (*p* < 0.0001) (Figure 5B).

## DISCUSSION

Many cancers studies have reported that the principal mechanisms of the inactivation of tumor suppressor genes is the hypermethylation of promoter regions associated with gene silencing [10, 11]. In the last two decades, the roles of epigenetic modifications, particularly DNA methylation, in carcinogenesis and its progression have attracted increasing attention [12, 13]. In our present study, we sought to identify the pivotal genes and assess their prognostic values and molecular mechanism in lung AD. MethyLight method was used to check the methylation status in the promotor regions of sixteen loci in lung AD tissues and AdjNL tissues. The 16 loci (Table 1) chosen for the DNA methylation analysis have been demonstrated to be hypermethylated in lung or the other kinds of cancers, and all these genes play key roles in cell cycle modulation, DNA repair, transcription, and apoptosis, thus, for our study, we selected these loci for further analysis. Taken from our above results, three loci can much probably be taken as DNA hypermethylation markers for the early stage of lung AD. These loci are HOXA11, CDKN2A EX2 and EYA4. The ROC curve shows that the AUC is 0.83–0.91 for the top three genes as follows: HOXA11 (AUC = 0.91), CDKN2A EX2 (AUC = 0.90), and EYA4 (AUC = 0.83). The HOXA11 and CDKN2A genes appear to be highly promising DNA hypermethylation markers for the development of non-invasive molecular markers of lung AD. These results are consistent with previous findings [14, 15]. Then, we chose these three genes for the further study of their mRNA and protein expression. Interestingly, we found that the HOXA11 protein was significantly higher in AIS than that in AD. This result has not yet been reported in the literature. Our results indicated that HOXA11 could serve as a potential therapeutic target and predicts patients’ prognosis after a surgical operation.

Homeobox sequences contain a helix-turn-helix DNA binding motif and encode 60-amino-acid homeodomain [16]. The HOXA11 gene is part of the cluster named A on chromosome 7, which is a region frequently deleted in human cancers. A DNA-binding transcription factor modulating gene expression, differentiation and morphogenesis was encoded in HOXA11 protein, and this gene played important roles in the development of uterine and female fertility [17]. HOXA11 is hypermethylated in lung AD even in the early stage IA tissues [18]. Similarly, here, we demonstrate that HOXA11 is hypermethylated in lung AD. Moreover, in the present study, the AIS samples were from the patients who had 100% 5-year survival rate, in another study had gotten the survival rate of AD is about 15% [2]. So our result showed that the down-regulated expression of the HOXA11 gene was associated with worse survival rates.

Finally, we analyzed the effect of HOXA11 hypermethylation on patient prognosis. We investigated the HOXA11 protein expression by IHC using samples from lung AIS and lung AD patients. Our results showed that the down–regulated expression of the HOXA11 gene could represent an independent unfavorable prognostic molecular marker for lung AD. This study supported that HOXA11 might function as a tumor suppressive gene in lung AD. Research by Mericskay M. et al. showed that a potential mechanism by which the HOXA11 gene is regulated by the formation of a regulatory loop between Wnt5a and Wnt7a in the mammalian female reproductive tract (FRT) [19]. Therefore, we hypothesized that the HOXA11 gene could be regulated by the formation of a loop between Wnt5a and Wnt7a in human lung cancer. In the further study, we intend to elucidate the exact regulatory mechanism and probably signaling pathways involved of the HOXA11 gene in human lung cancer.

In summary, our results showed that the promotor regions of HOXA11, CDKN2A EX2 and EYA4 were frequently methylated in human lung AD tissues. HOXA11 and CDKN2A EX2 could be confirmed as DNA hypermethylation markers for lung AD. The expression of HOXA11 mRNA and protein was regulated by DNA methylation in promoter region in human lung tumor cell lines and human lung AIS and AD tissues. HOXA11 was a new valuable factor for predicting recurrence and survival in lung AD after surgical resection in clinical settings.

## MATERIALS AND METHODS

### Patients and tissue specimens

All samples were obtained from the human tumor bank at the Institute of Health Sciences, Shanghai Institutes for Biological Sciences. The tissues were bisected, one sample was collected, immediately snap-frozen in liquid nitrogen and then stored at −80°C for further analysis; the other samples were fixed in 4% buffered formaldehyde and embedded in paraffin for further use. This study was approved by the institutional biomedical research ethics committee of the Shanghai Institutes for Biological Sciences (Chinese Academy of Sciences), and written informed consent was obtained from each patient. All cases were diagnosed histologically according to the World Health Organization classification [20] and the Noguchi classification [21] for small-sized AD.

### Genomic DNA extraction and bisulfite treatment

In total, 21 pairs of formaldehyde fixed paraffin blocks of lung AD and AdjNL tissues and 2 NL tissues were used for the MethyLight analysis. The patients’ ages ranged from 45–82 years at the time of surgery (median: 65 years old). Hematoxylin and eosin-stained slides were reviewed by an experienced pathologist to confirm the original classification of the tumor and mark the lesions to be retrieved. DNA was extracted from manually microdissected tumor and non-tumor lung samples under a microscope via proteinase K digestion [22]. Briefly, the cells were lysed in a solution containing 100 mmol/L Tris-Hcl (pH 8.0), 10 mmol/L EDTA (pH 8.0), 1 mg/mL proteinase K, and 0.05 mg/mL tRNA and incubated at 50°C overnight. The bisulfate conversion was completed with the EZ DNA methylation^TM^ kit (#5001) (Zymo Research Corporation, CA. USA.) according to the manufacturer's instructions [23]. The bisulfite-treated DNA was subjected to quality control tests to determine the DNA amount and bisulfate conversion [9].

### DNA methylation analysis

The DNA methylation analysis was performed using MethyLight [24]. The primer and probe sequences are listed in Supplementary Table 1. In addition to the primers and probe sets designed specifically for the genes of interest, an internal reference primer and probe set designed to analyze Alu repeats (Alu) were included in the analysis to normalize the input DNA [25]. The percentage methylated reference (PMR) was calculated as the [GENE *X*] _sample_/ [control] _sample_ divided by [GENE *X*] _SssI_/ [control] _SssI_ (SssI treated human white blood DNA) and multiplied by 100.

### Cell culture

Eight human lung cancer cell lines (95-D, A549, NCI-H292, NCI-H460, NCI-H661, NCI-H1299, LTEP-a-2, and SPC-A-1) were purchased from the Type Culture Collection of the Chinese Academy of Sciences, Shanghai, China. All cell lines were grown in RPMI-1640 (HyClone) supplemented with 10% fetal bovine serum (FBS) (GIBCO) and 100 U/ml penicillin and streptomycin (P/S). All cell lines were incubated at 37°C with 5% CO2.

### 5-Aza-2′-deoxycytidine treatment

The lung AD cell lines 95-D and A549 were treated with a culture medium containing a demethylating agent, 5-aza-2′-deoxycytidine (5-Aza-dC; Sigma-Aldrich, USA). 5-Aza-dC was dissolved in dimethyl sulfoxide (DMSO). The 95-D and A549 cells (1 × 10^6^ cells/100 mm dish) were incubated in a culture medium with 5-Aza-dC (final concentration of 2 μmol/L) and without 5-Aza-dC (DMSO; final concentration of 2 μmol/L) for 48 hrs. The culture medium was changed every day. After harvesting the cells, RNA was extracted as described below for reverse transcription Quantitative real-time-PCR (RT-qPCR), and protein was extracted for the Western blotting analysis.

### RNA isolation and RT-qPCR

In total, 30 pairs of frozen lung AD tissues and adjacent non-tumor tissues were used to detect the expression of the HOXA11, EYA4 and CDKN2A EX2 genes by RT-qPCR. Total RNA was isolated with TRIzol Reagent (Invitrogen/Thermo Fisher Scientific, Waltham, MA) according to the manufacturer's instructions, and the samples were stored at −80°C until use. Reverse transcription was performed using the PrimeScript RT Reagent Kit (TaKaRa, Shiga, Japan). Quantitative PCR was carried out using the SYBR Green PCR Master Mix (TaKaRa) in the ABI Prism7900 (Applied Biosystems/Thermo Fisher Scientific, Waltham, MA). All gene expression results were normalized to the expression of the housekeeping gene GAPDH. The PCR primers are shown in Supplementary Table 2.

### Protein extraction and western blotting analysis

Protein samples from the cell lines and tissues were prepared with a lysis buffer, M-PER (PIERCE Biotechnology, Rockford, IL, USA), containing proteinase inhibitors. Total protein aliquots were mixed with the Laemmli sample buffer, denatured at 95°C for 5 min and separated on a 10% Tris-HCl gel (Bio-Rad). The proteins were transferred to polyvinylidene difluoride membranes and blocked with 5% (w/v) nonfat milk/TBST for 1 h at room temperature. The membranes were incubated overnight at 4°C with primary antibodies directed against HOXA11 (1:5000, ab28699, Abcam, USA) in 5% BSA/TBST (w/v). The membranes were washed three times with TBST and incubated with an HRP-linked secondary antibody for 1 h at room temperature. The protein bands were visualized using electro-chemiluminescence (ImageQuant LAS4000, USA). The anti-Tubulin antibodies served as the loading control.

### Immunohistochemistry of formalin-fixed tissue microarray (TMA) sections

Paraffin-embedded TMA blocks, including 160 lung adenocarcinoma samples (40 lung AIS, 40 lung AD and 80 normal counterparts), were sectioned at a 4 μm thickness, deparaffinized in xylene, and rehydrated in graded ethanol solutions, and the endogenous peroxidase activity was blocked by incubation with 3% H_2_O_2_ for 30 min at room temperature. Then, the sections were immersed in a citrate-NaOH buffer (10 mM sodium citrate, pH 7.0) for 40 min at 92°C to restore antigenicity. The rehydrated sections were incubated overnight at 4°C with the rabbit anti-human HOXA11 polyclonal antibody (1:250, ab28699, Abcam, USA.). The sections incubated with the first antibody were washed with Tris-buffered saline (TBS) and were then incubated with the MaxVision ^TM^ HRP-Polymer anti-Rabbit IHC Kit (Maixin, Fuzhou, China) for 15 min at room temperature. The sections were visualized using the DAB Detection Kit (Maixin, Fuzhou, China), and the reaction was followed by counterstaining with hematoxylin. The negative control experiments were performed by omitting the primary antibody.

### Scoring of HOXA11 expression

The evaluation of the immunohistochemical staining was performed independently by two authors without knowledge of the clinicpathological information. The immunoreactive scores of HOXA11 were determined by the sum of extension and intensity. The intensity of the staining was scored using the following scale: 0, no staining; +, mild staining; ++, moderate staining and +++, marked staining. The area of staining was evaluated and recorded as a percentage: 0, less than 5%; +, 5%–25%; ++, 26%–50%; 3+, 51%–75% and 4+, more than 75%. The combined scores were recorded and graded as follows: -, 0; +, 1–2; ++, 3-5; +++, 6–7.

### Statistical analyses

All statistical analyses used in this paper are two side *T* test. *p* < 0.05 shows significant difference. Receiver operating characteristic (ROC) curves were plotted using the AD vs. AdjNTL lung PMR value by SPSS software (SPSS Inc., Chicago, IL, USA). The two-dimensional hierarchical clustering of samples and methylated genes were analyzed by the R programming language (R 3.0.1).

## SUPPLEMENTARY MATERIALS TABLES


