# Supplementary material for: DNA methylation profiling identifies the HOXA11 gene as an early diagnostic and prognostic molecular marker in human lung adenocarcinoma
Source: Oncotarget. 2017 Mar 23;8(20):33100–9. doi: 10.18632/oncotarget.16528 (PMC5464853; doi:10.18632/oncotarget.16528)
Supplement: Supplementary file 1 [file oncotarget-08-33100-s001.pdf]

DNA methylation profiling identifies the HOXA11 gene as an early diagnostic and prognostic molecular marker in human lung adenocarcinoma

Supplementary Materials

Supplementary Table 1: Primers and probes of 16genes

Official symbol	Forward Primer Sequence	Reverse Primer Sequence	Probe Oligo Sequence
CDH13	AATTTCGTTCGTTTTGTGCGT	CTACCCGTACCGAACGATCC	AACGCAAAACGCGCCCGACA
CDKN2A	GCGTTCGAGTGGCGGA	CTCCCGAACAACGTCGTACAC	CAATTAAACTCCGCGCCGTAAAACAACAA
CDX2	GGTAATCGTCGTAGTTCGGGTATT	ACTCCGTACGCCACTCTAACG	CAACCTAACGCCGCAAAACTTCGTCA
EYA4	TGGATAGGATGGAAGTTTTGCG	AACTACCGACAACGCGACG	CGCTCCGACCGTTCCCGACTT
HOXA1	GTTGTTGCGGCGATTGTAAA	CGCGCAAAACGCAACTT	TACTCTTCTTCGCTCCAACACTCCAAATCG
HOXA11	TTTTGTTTTCGATTTTAGTCGGAAT	TAATCAAATCACCGTACAAATCGAAC	ACCACCAAACAAACACATCCACGACTTCA
NEUROD1	GTTTTTTGCGTGGGCGAAT	CCGCGCTTAACATCACTAACTAAA	CGCGCGACCACGACACGAAA
NEUROD2	GGTTTGGTATAGAGGTTGGTATTTCGT	ACGAACGCCGACGTCTTC	CGCCATACGAACCGCGAAACGAATATAA
ONECUT2	CGTTACGTATATCGCGCGG	CAAAAACCTCCTATATAAACGACGAAT	AACACGCAATTACGCGCTTTTATACGCA
OPCML	CGTTTCGAGGCGGTATCG	CGAACCGCCGAAATTATCAT	AACAACTCCATCCCTAACCGCCACTTTCT
PTPRN2	CGTTTTAATAGTTTCGGGTTTAGTTATAAGT	AACTACGCTTTCTCAACGCCTC	TAAAACGACCGCGTACTCGCCAAAAAA
RASSF1	ATTGAGTTGCGGGAGTTGGT	ACACGCTCCAACCGAATACG	CCCTTCCCAACGCGCCCA
SFRP1	GAATTCGTTCGCGAGGGA	AAACGAACCGCACTCGTTACC	CCGTCACCGACGCGAAAACCAAT
TMEFF2	CGACGAGGAGGTGTAAGGATG	CAACGCCTAACGAACGAACC	TATAACTTCCGCGACCGCCTCCTCCT
TWIST1	GTAGCGCGGCGAACGT	AAACGCAACGAATCATAACCAAC	CCAACGCACCCAATCGCTAAACGA

Supplementary Table 2: Primer sets used for qRT-PCR

Official symbol	Forward Primer Sequence	Reverse Primer Sequence
CDKN2A	ATATGCCTTCCCCCACTACC	CGTGAGTGCTCACTCCAGAA
EYA4	AATTACGGACGCACTGAACC	CTCACTTCTCCTGCCACTCC
HOXA11	TCCCATTGAATCTCCTTTGC	ATTTTCCTTGTGCCCAGTTG
